# Supplementary material for: Diagnosis and Characterization of Plant Viruses Using HTS to Support Virus Management and Tomato Breeding
Source: Viruses. 2024 May 31;16(6):888. doi: 10.3390/v16060888 (PMC11209215; doi:10.3390/v16060888)

Sample JCM07-5

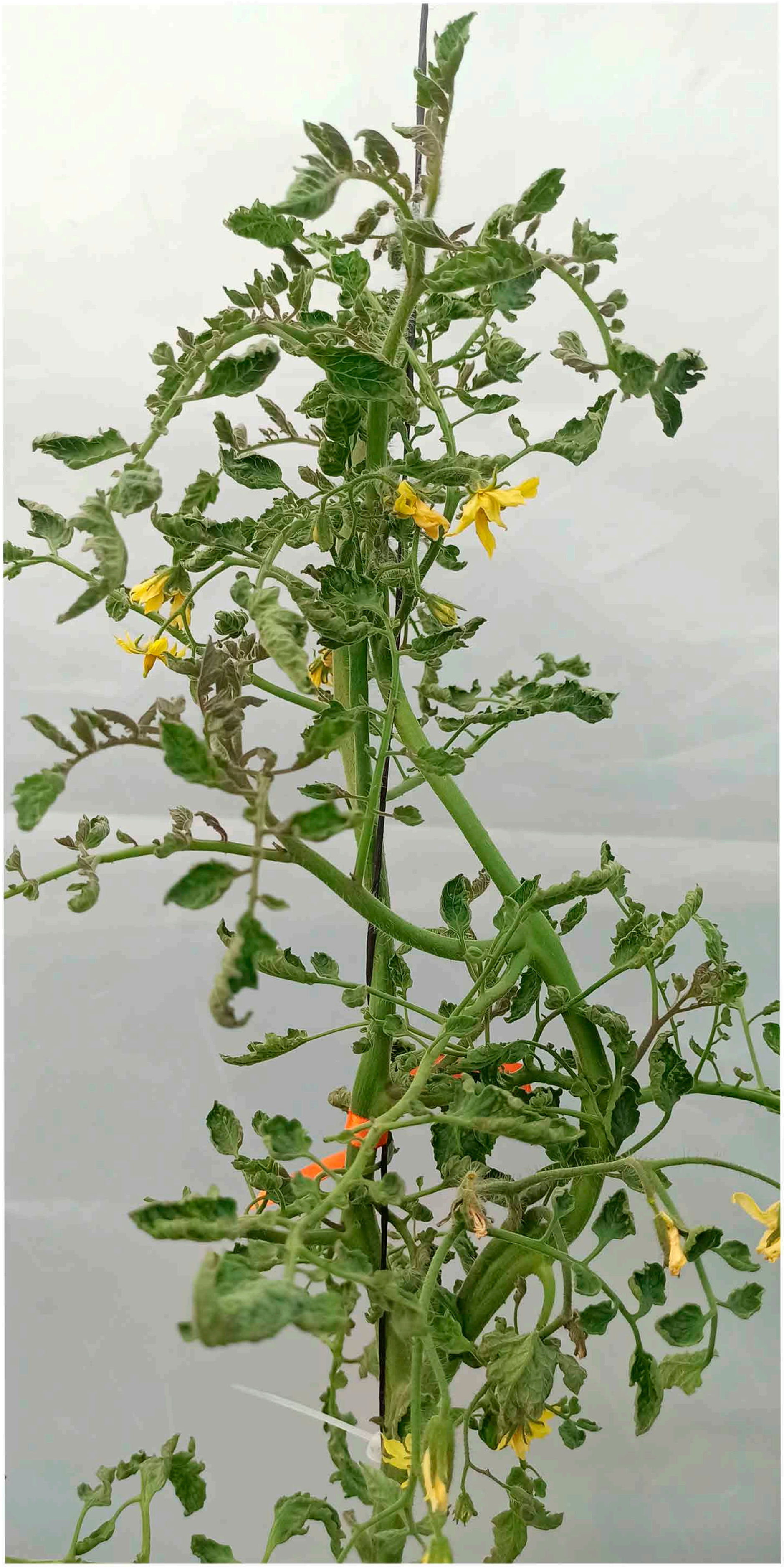

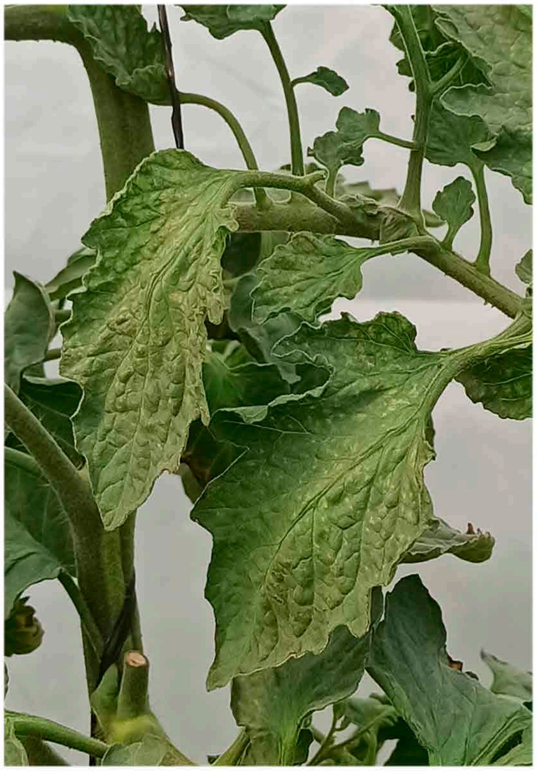

PHYVV + ToMV

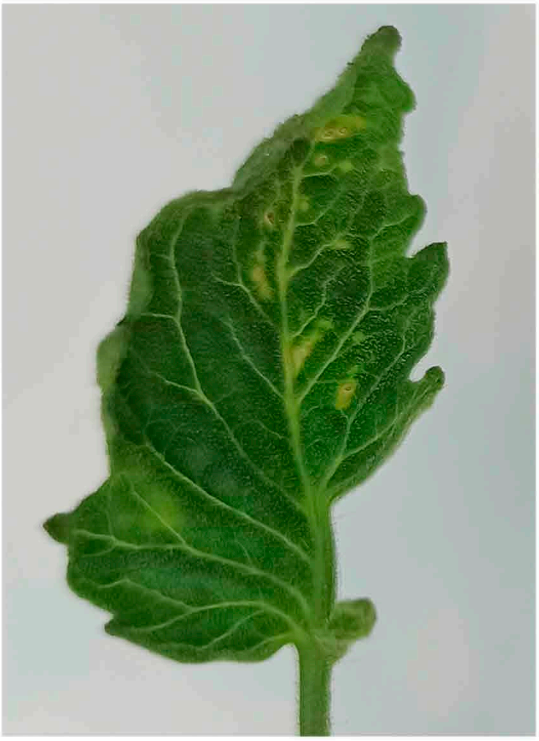

Sample JCM08-2

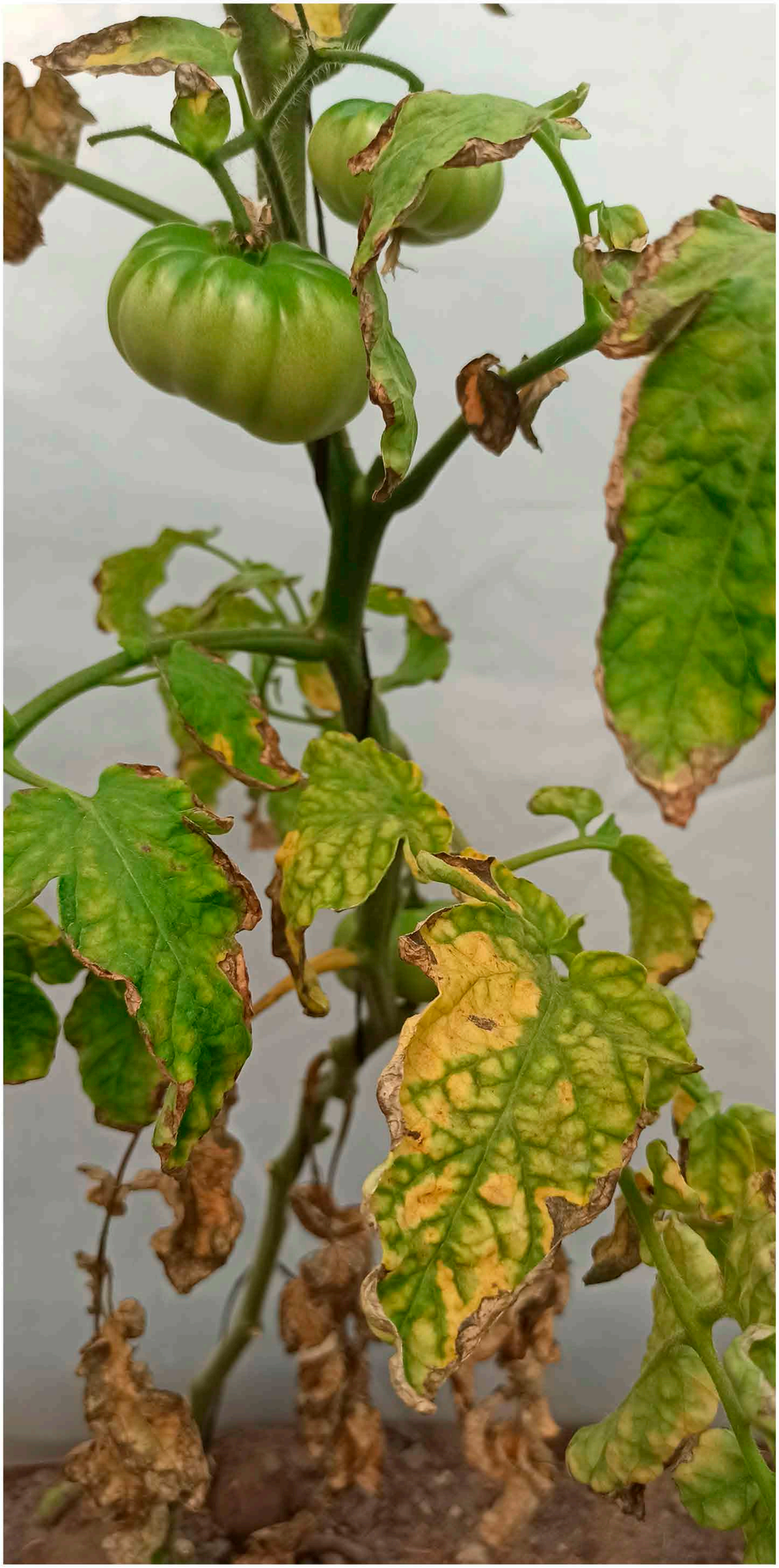

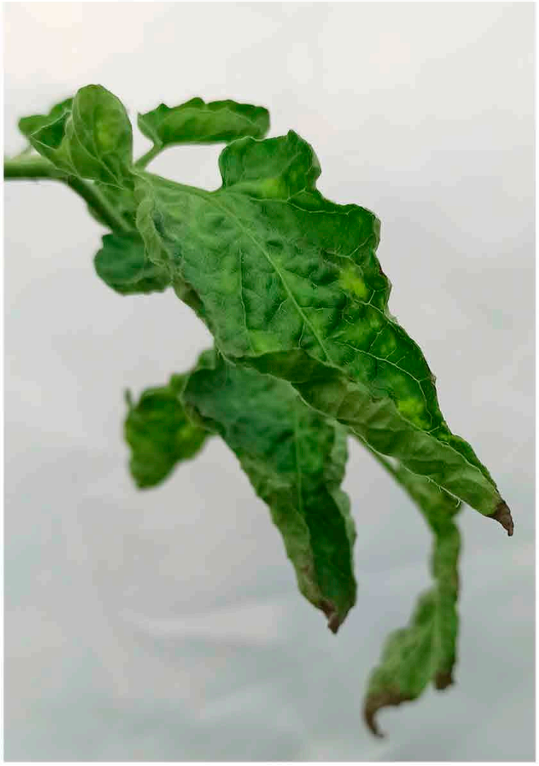

ToGMoV + ToMV

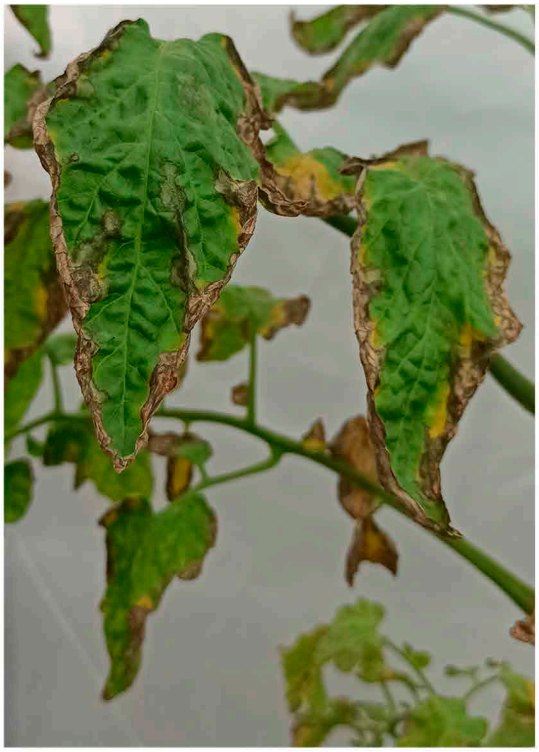

Sample JCM08-3

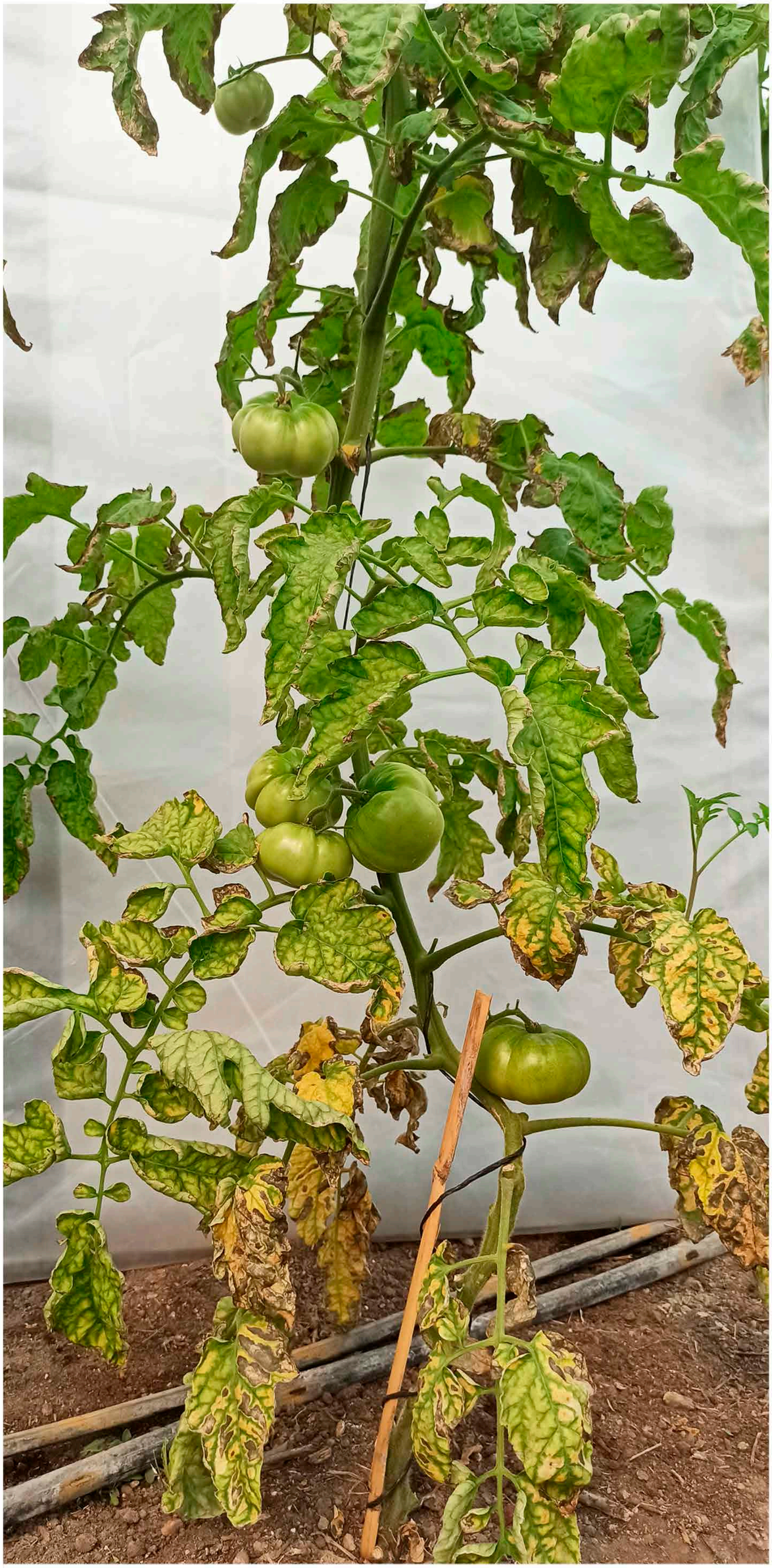

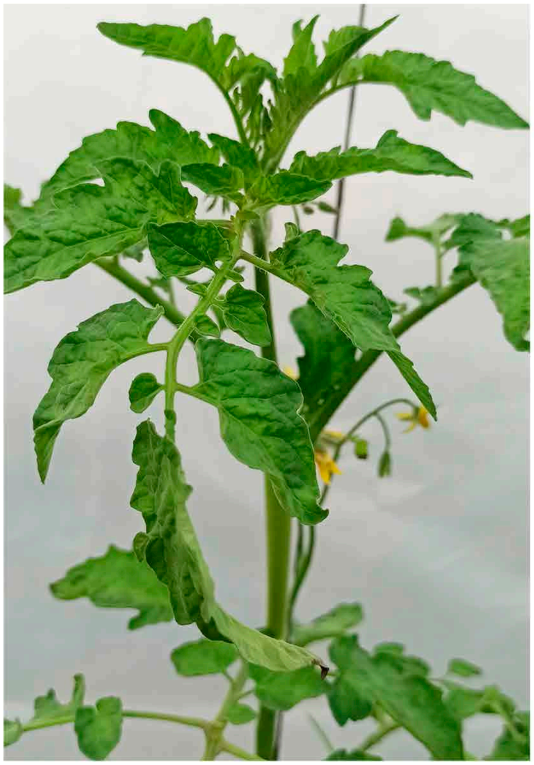

ToGMoV + ToMV

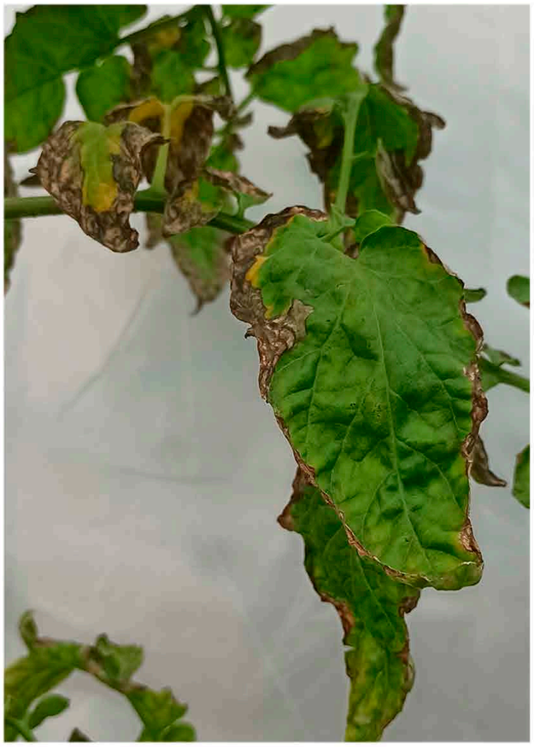

Sample JCM07-1

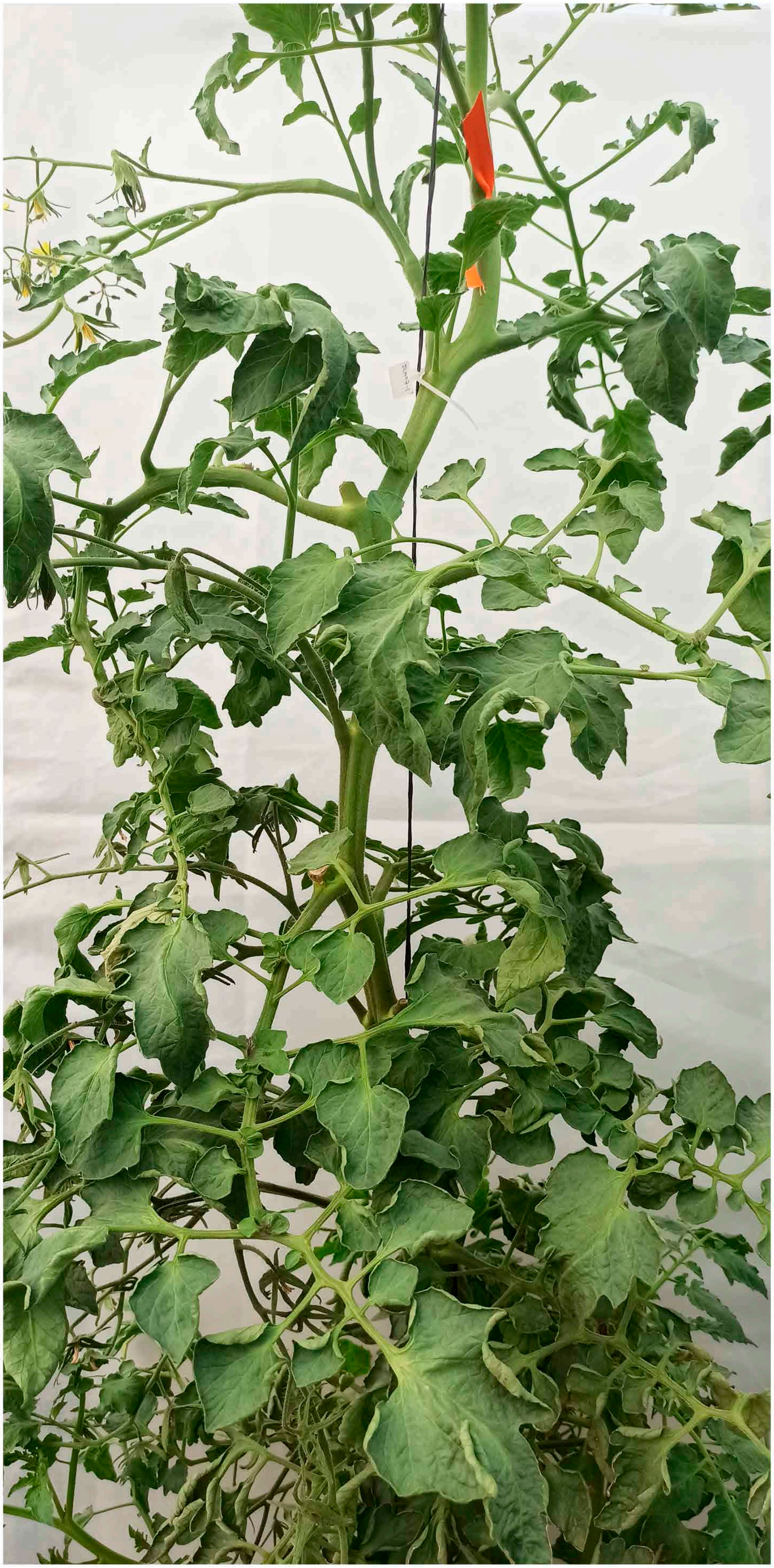

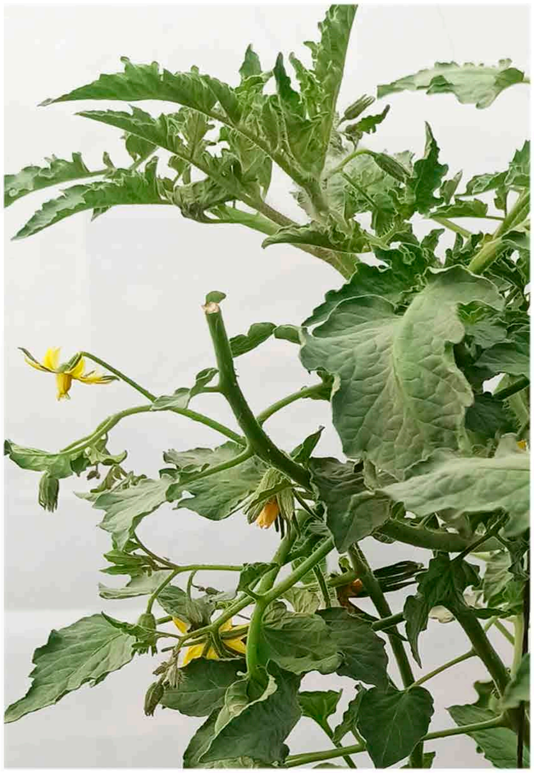

ToMV

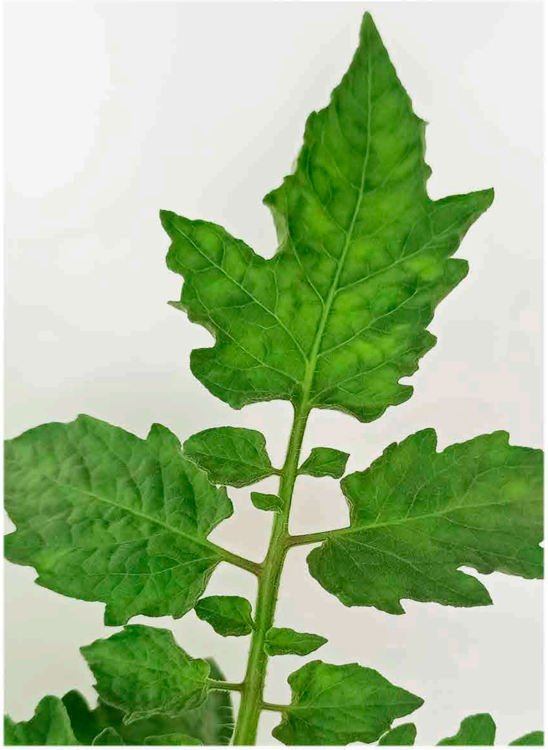

Sample JCM07-11

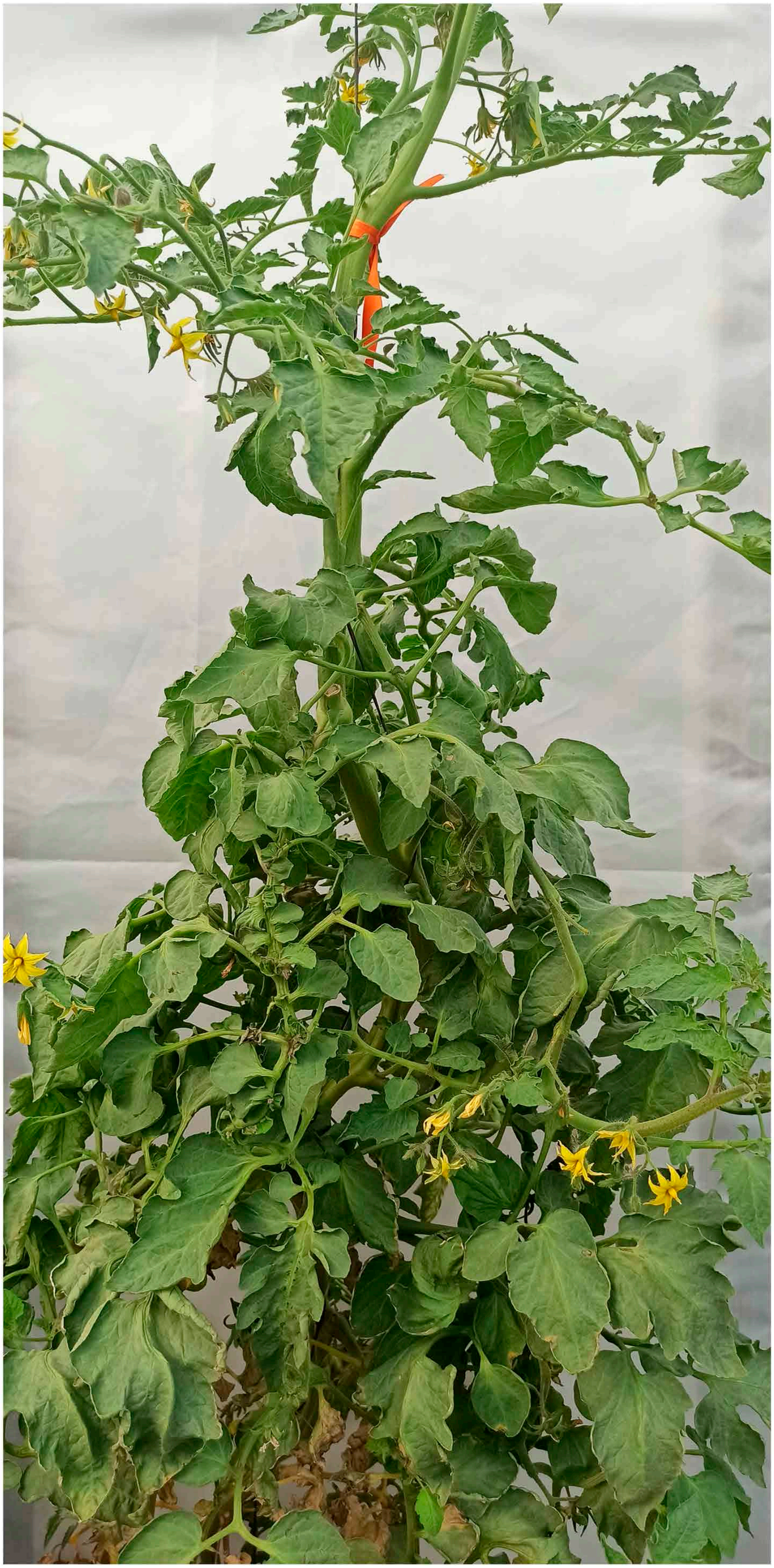

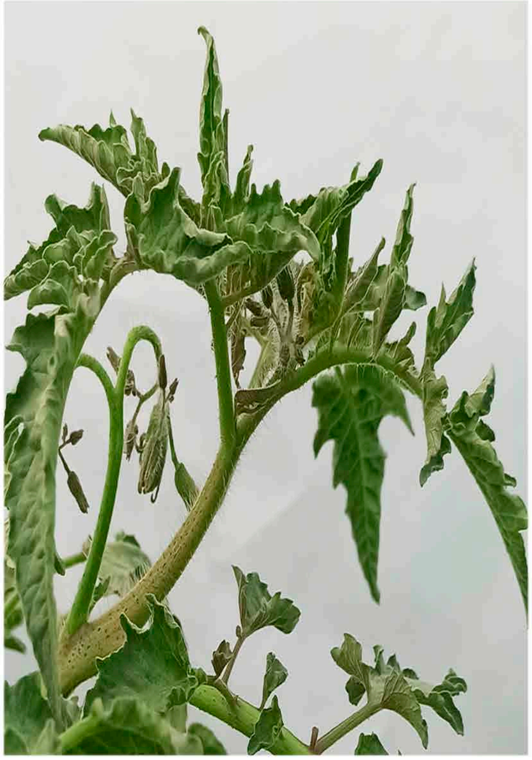

ToMV

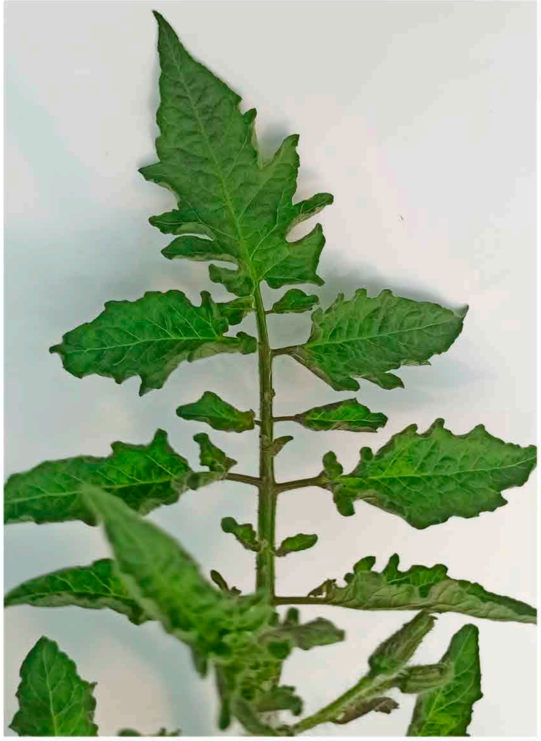

Supplement: Supplementary file 1 [file viruses-16-00888-s001.zip › Supplementary materials/Figure S1.pdf]
