# Supplementary material for: Diagnosis and Characterization of Plant Viruses Using HTS to Support Virus Management and Tomato Breeding
Source: Viruses. 2024 May 31;16(6):888. doi: 10.3390/v16060888 (PMC11209215; doi:10.3390/v16060888)
Supplement: Supplementary file 1 [file viruses-16-00888-s001.zip › Supplementary materials/Figure S3.pdf]

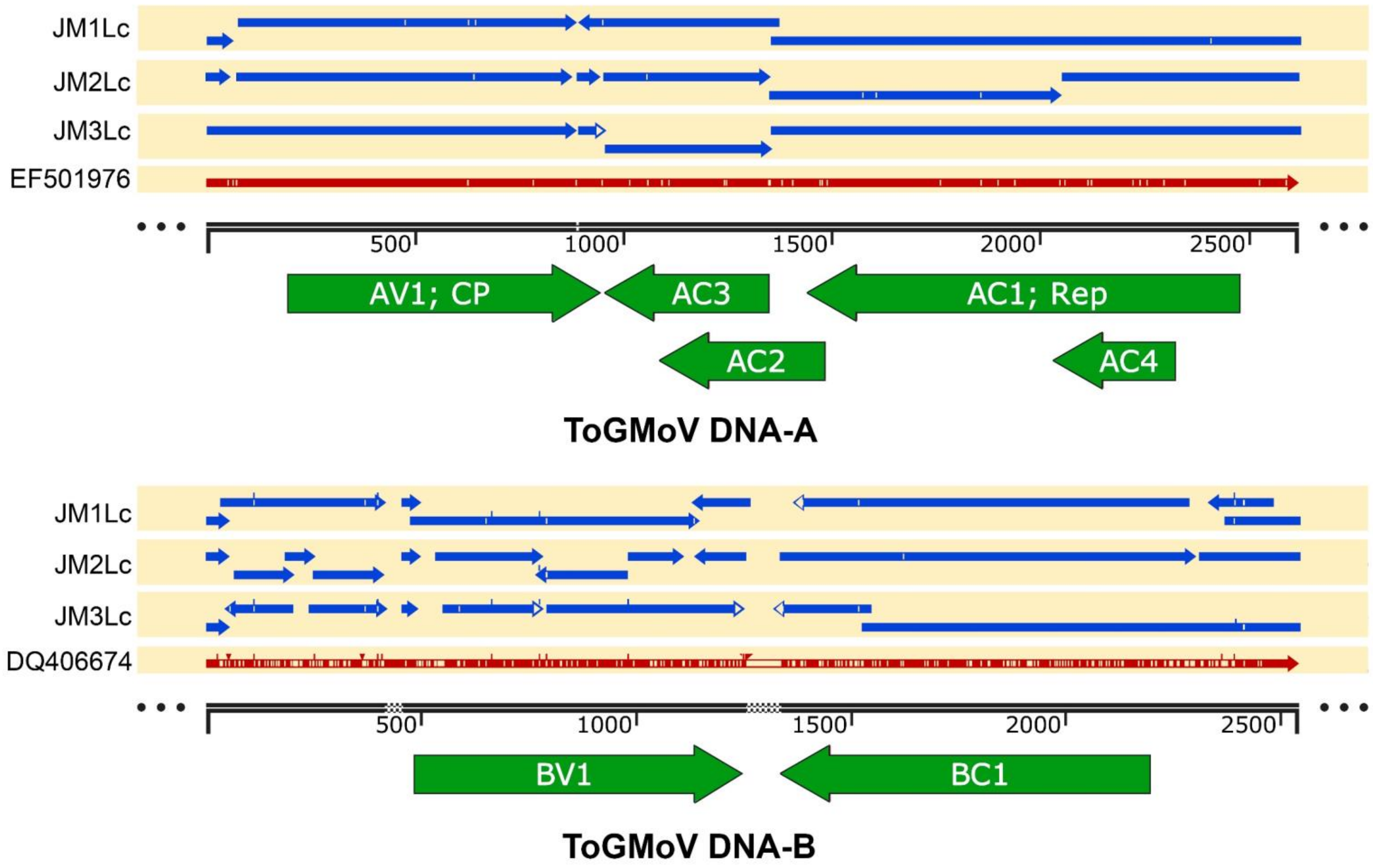

Figure S3. Schematic representation of the coverage of assembled contigs across the viral reference genome and the reconstructed genome sequences of ToGMoV DNA-A and DNA-B components. The library name and NCBI accession number of the viral reference genome sequence are indicated on the left. Contigs are shown in blue and putative open reading frames (ORFs) in green. The black line represents the reconstructed genome sequence, while the dotted line corresponds to the region where no sequence was covered by the assembled contigs.
