# Supplementary material for: Diagnosis and Characterization of Plant Viruses Using HTS to Support Virus Management and Tomato Breeding
Source: Viruses. 2024 May 31;16(6):888. doi: 10.3390/v16060888 (PMC11209215; doi:10.3390/v16060888)
Supplement: Supplementary file 1 [file viruses-16-00888-s001.zip › Supplementary materials/Figure S4.pdf]

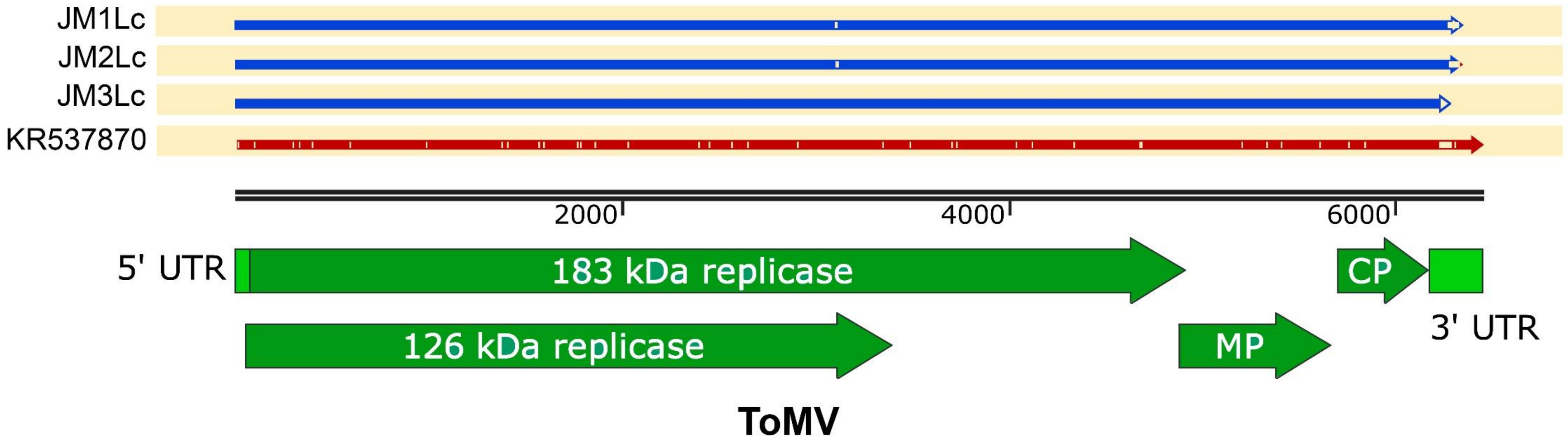

Figure S4. Schematic representation of the coverage of assembled contigs across the viral reference genome and the reconstructed genome sequences of ToMV. The library name and NCBI accession number of the viral reference genome sequence are indicated on the left. Contigs are shown in blue and putative open reading frames (ORFs) in green. The 3′ UTR sequence was determined by RT-PCR and Sanger sequencing. The black line represents the reconstructed genome sequence.
